# Cinnamophilin ameliorates testosterone-induced prostatic hyperplasia and fibrosis by regulating 5α-reductase and TGF-β/Smad signaling pathway

**DOI:** 10.22038/ijbms.2025.88921.19197

**Published:** 2026

**Authors:** Di Han, Chung-Yi Chen, XiangPeng Huang, Yi Liu, Hui Sun, YiDan Li, ManYu Liao, JiaYi Cai, Jing Liu, WenHui Li, Peng Zhang, ZhengPing Wu, Chi-Ming Liu

**Affiliations:** 1 College of Basic Medical Sciences, Yichun University, Yichun, Jiangxi Province 336000, China; 2 College of Chemistry and Bio-engineering, Yichun University, Yichun, Jiangxi Province 336000, China; 3 School of Medical and Health Sciences, Fooyin University, Daliao, Kaohsiung 83102, Taiwan; 4 School of Clinical Medicine, Yichun University, Yichun, Jiangxi Province 336000, China

**Keywords:** Cinnamophilin, EMT, Fibrosis, Prostate hyperplasia, Smad, TGF-β

## Abstract

**Objective(s):**

Androgen and TGF-β1/Smad signaling pathways play important roles in epithelial-mesenchymal transition (EMT), fibrosis, and the development of benign prostatic hyperplasia (BPH). Cinnamophilin is extracted from *Cinnamomum philippinense*. The anti-proliferative and anti-fibrosis effects of cinnamophilin on the prostate remain unclear. This study aimed to investigate the therapeutic effects and molecular mechanism of action of cinnamophilin on prostate growth in testosterone propionate (TP)-treated mice.

**Materials and Methods:**

The study was conducted both *in vivo* and *in vitro*. TP was injected subcutaneously to induce prostate enlargement and growth. Cinnamophilin (40 mg/kg) was orally administered once a day in TP (7.5 mg/ kg)-treated mice for 28 days. The morphological characteristics and fibrosis of the prostate were examined by H&E (Hematoxylin and Eosin) and Masson’s trichrome stain. Protein expression was determined by Western blot. BPH-1 and WPMY-1 cells were treated with different concentrations of cinnamophilin (1–100 μM).

**Results:**

Cinnamophilin (40 mg/kg) significantly reduced prostate weight and prostate index in animal models. Cinnamophilin inhibited the protein expression of 5α-reductase type II and prostate-specific antigen (PSA) in TP-treated mice. Cinnamophilin reversed morphological changes, EMT, and fibrosis in TP-treated mice. Cinnamophilin increased E-cadherin but decreased N-cadherin, vimentin, fibronectin, α-SMA, TGFBR2, TGF-β1, p-Smad2/3, collagen I, collagen III, and collagen IV protein expressions. The expression of Smad2/3 was not significantly different among these groups. Cinnamophilin (100 μM) inhibited proliferation at 48 hr in BPH-1 and WPMY-1 cells.

**Conclusion:**

These findings suggest that cinnamophilin inhibits prostate growth and mitigates EMT and fibrosis by regulating TGFβ/Smad signaling pathways.

## Introduction

Benign Prostatic Hyperplasia (BPH) is a prevalent clinical issue among older men (1), characterized by the abnormal proliferation of epithelial, stromal, and smooth muscle cells in the prostate, leading to lower urinary tract symptoms (LUTS) (2). Men over the age of 45 develop BPH, and the prevalence is about 80% in men over 70 (3). A previous study reported that 94 million men suffered from BPH in 2019 (4). Factors such as age, oxidative stress, inflammation, growth factors, and sex hormones contribute to BPH development (2, 5, 6). Among these, sex hormones play a critical role. The enzyme 5α-reductase converts testosterone (T) into dihydrotestosterone (DHT), which binds to the androgen receptor (AR) to regulate gene expression and prostate growth. Consequently, 5α-reductase inhibitors are widely used **for **BPH treatment. Additionally, phytotherapy is an alternative treatment, such as the saw palmetto (*Serenoa repens*) fruit extract, for BPH (7).

Epithelial-mesenchymal transition (EMT) is involved in embryogenesis, metastasis, fibrosis, and BPH (8-10). EMT is characterized by the reduction of epithelial markers, such as E-cadherin, and the up-regulation of mesenchymal markers, including N-cadherin and vimentin. Transforming growth factor-β (TGF-β) is a key cytokine that mediates EMT and fibrosis, contributing to BPH progression. Fibrosis results from an abnormal response to organ injury, involving fibroblast and myofibroblast proliferation, along with excessive extracellular matrix (ECM) production and deposition mediated by TGF-β (11). Chronic prostatic inflammation exacerbates fibrosis, further aggravating LUTS (12). Therefore, reversing EMT and fibrosis represents a promising therapeutic strategy for BPH.

The Cinnamomum genus has many pharmacological activities and is widely used in Asia (13). Cinnamophilin, a lignan compound isolated from *Cinnamomum philippinense*, possesses various pharmacological activities, including thromboxane synthase inhibition, thromboxane A2 (TXA2) receptor blockade, free radical scavenging, and anti-oxidation (14, 15). Cinnamophilin inhibited NADPH-dependent microsomal lipid peroxidation, possessed free radical scavenging capacity, and protected rat aortic smooth muscle cells from free radical-induced cell damage (16). Recently, cinnamophilin has been found to enhance temozolomide-induced cytotoxicity by suppressing the ROS production and cell cycle arrest in glioma cell lines (17).

BPH is an abnormal proliferation of prostate cells. Although the pharmacological mechanism of action of cinnamophilin in BPH remains unclear, the anti-proliferative effects of cinnamophilin are demonstrated in glioma cells. The overproduction of ROS and up-regulation of AR signaling are involved in BPH pathogenesis (18). This study aimed to explore the potential signaling pathways, molecular targets, and determine the anti-proliferation activity of cinnamophilin in counteracting testosterone-induced prostate enlargement. The therapeutic efficacy of cinnamophilin against (TP)-treated mice and BPH-1 and WPMY-1 cells was also examined in this study.

## Materials and Methods

### Chemicals and reagents

Finasteride (cat. no. F156753), testosterone propionate (cat. no. T101368), and cinnamaldehyde (cat. no. C108630) were purchased from Aladdin Biochemical Technology Co., Ltd (Shanghai, China). Dulbecco’s Modified Eagle Medium (DMEM) and RPMI-1640 medium, both supplemented with 10% fetal bovine serum (FBS) and penicillin-streptomycin, were obtained from Wuhan Servicebio Technology Co., Ltd. Cinnamophilin was a gift from Professor Chung-Yi Chen (Fooyin University, Kaohsiung, Taiwan) and extracted and identified in a previously published study (19).

### Animal experiments

Seven-week-old male Institute of Cancer Research (ICR) mice were obtained from Hunnan SJA Laboratory Animal Co., Ltd. The mice were accommodated in a 12-hour light/dark cycle at 22 ± 2 °C. All procedures adhered to the guidelines of the Animal Care and Ethics Committee of Yichun University (Approval No. 2023029). Cinnamophilin, cinnamaldehyde, testosterone propionate (TP), and finasteride were dissolved in corn oil. Finasteride, a 5α-reductase inhibitor, was used as a positive control. Cinnamaldehyde is a well-known bioactive compound extracted from species of the genus Cinnamomum. In the current study, the effects of cinnamaldehyde and cinnamophilin are compared in TP-induced mice. Cinnamophilin, cinnamaldehyde, and finasteride were administered orally via nasogastric tube once daily, while TP was administered subcutaneously once daily for 28 days. The TP-induced prostate growth method followed that of a previous study (20). Mice were randomly divided into five groups (n = 6 per group): (A) control, (B) testosterone propionate model (TP, 7.5 mg/ kg), (C) TP + cinnamophilin (40 mg/kg), (D) TP + cinnamaldehyde (40 mg/ kg), and (E) TP + finasteride (5 mg/kg). Mice were weighed weekly. Prostates were collected 24 hr after the final treatments. The mice were euthanized via cervical dislocation, and the prostates were immediately removed, cleaned, weighed, and used to calculate the prostate index: Prostate index = prostate weight of mice (g)/ body weight of mice (g) ×1000.

### Histopathological examination

Prostatic specimens were fixed in 10% formalin overnight, embedded in paraffin, and sectioned into 4-mm-thick slices. Sections were stained with hematoxylin and eosin (H&E) and Masson’s trichrome stain (20). Images were captured using a 10x40 light microscope (Nikon TI-DH) and analyzed with NIS-Elements software (version 4.30, Nikon). 

### Western blot analysis

Proteins were extracted from mouse prostate tissues by using T-PER (cat. no. 78510; Thermo Fisher Scientific, Inc., Massachusetts, USA) with protease inhibitors. Protein concentrations were quantified using the Pierce Bradford Protein Assay Kit (cat. no. 23200; Thermo Scientific, Inc.). Equal amounts (20 μg) of protein per lane were loaded and separated by SDS-PAGE, transferred to PVDF membrane (Immobilon^®^-P PVDF Membrane, cat. no. IPVH00010; MilliporeSigma, Massachusetts, USA), and blocked with 5% skim milk for 1 hr at room temperature. Membranes were incubated overnight at 4 °C with primary antibodies, including β-actin (1:8000 dilution; cat. no. A5441; MilliporeSigma, Massachusetts, USA), androgen receptor (1:1000 dilution; cat. no. CY5030; Abways Technology, Shanghai, China), TGF-β1 (1:1000 dilution; cat. no. CY2179; Abways Technology), fibronectin (1:1000 dilution; cat. no. CY9537; Abways Technology), and 5α-reductase type II (1:1000 dilution; cat. no. CY8576; Abways Technology), E-cadherin (1:1000 dilution; cat. no. 3195; Cell signaling technology, Shanghai, China), TGFBR2 (1:1000 dilution; cat. no. AF5449; Affinity Biosciences LTD, Shanghai, China), phospho-Smad2/3 antibody (1:1000 dilution; cat. no. AF3367; Affinity Biosciences LTD), Smad2/3 (1:1000 dilution; cat. no. AF6367; Affinity Biosciences LTD), N-cadherin (1:1000 dilution; cat. no. AF5239; Affinity Biosciences LTD), vimentin (1:1000 dilution; cat. no. AF7013; Affinity Biosciences LTD), PSA (1:1000 dilution; cat. no. AF0246; Affinity Biosciences LTD), α-SMA (1:1000 dilution; cat. no. BF9212; Affinity Biosciences LTD), collagen I (1:1000 dilution; cat. no. AF7001; Affinity Biosciences LTD), collagen III (1:1000 dilution; cat. no. AF5457; Affinity Biosciences LTD), or collagen IV (1:1000 dilution; cat. no. AF0510; Affinity Biosciences LTD). After the incubation, the membranes were washed three times with TBST and incubated with secondary antibodies, including anti-rabbit IgG HRP-linked antibody (1:1000 dilution; cat. no. 7074; Cell Signaling Technology) and anti-mouse IgG HRP-linked antibody (1:1000 dilution; cat. no. 7076; Cell Signaling Technology). Membranes were stripped with Western Blot Fast Stripping Buffer (cat. no. PS107; Epizyme Biotech; Shanghai, China) at room temperature for 20 min and reprobed with antibodies. Chemiluminescence was detected using an ultra-sensitive ECL chemiluminescent substrate (cat. no. BL523B, Biosharp, Anhui, China). Densitometry was performed by ImageJ 1.52a software (National Institutes of Health, USA). 

### Cell lines and cell culture

Immortalized normal prostate stromal cells (WPMY-1) and human BPH epithelial cells (BPH-1) were purchased from Shanghai Fuheng Biotechnology Co., Ltd. (Shanghai, China) and Wuhan Servicebio Technology Co., Ltd. (Wuhan, China). Cells were cultured in DMEM or RPMI-1640 medium supplemented with penicillin (100 U/ml), streptomycin (100 μg/ml), and 10% FBS at 37 °C in a humidified atmosphere with 5% CO_2_.

### MTT assay

Cells were seeded into 96-well plates at a density of 1 × 10^4^ cells per well. At 80% confluency, the cells were treated with different concentrations of cinnamophilin (1, 10, and 100 μM) for 48 hr. Following incubation, 10 μl of 5 mg/ ml MTT solution was added to each well and incubated for 4 h at 37 °C. The medium was then replaced with 100 μl DMSO to dissolve formazan crystals at room temperature for 20 min. Absorbance was measured at 570 nm using a BIO-RAD microplate reader.

### Statistical analysis

All data are expressed as mean ± standard error (SE). Differences among groups were analyzed using one-way analysis of variance (ANOVA) followed by Tukey’s *post hoc* test. Statistical significance was set at *P*<0.05.

## Results

### Cinnamophilin inhibited TP-induced prostate growth in mice

**Figure 1:**
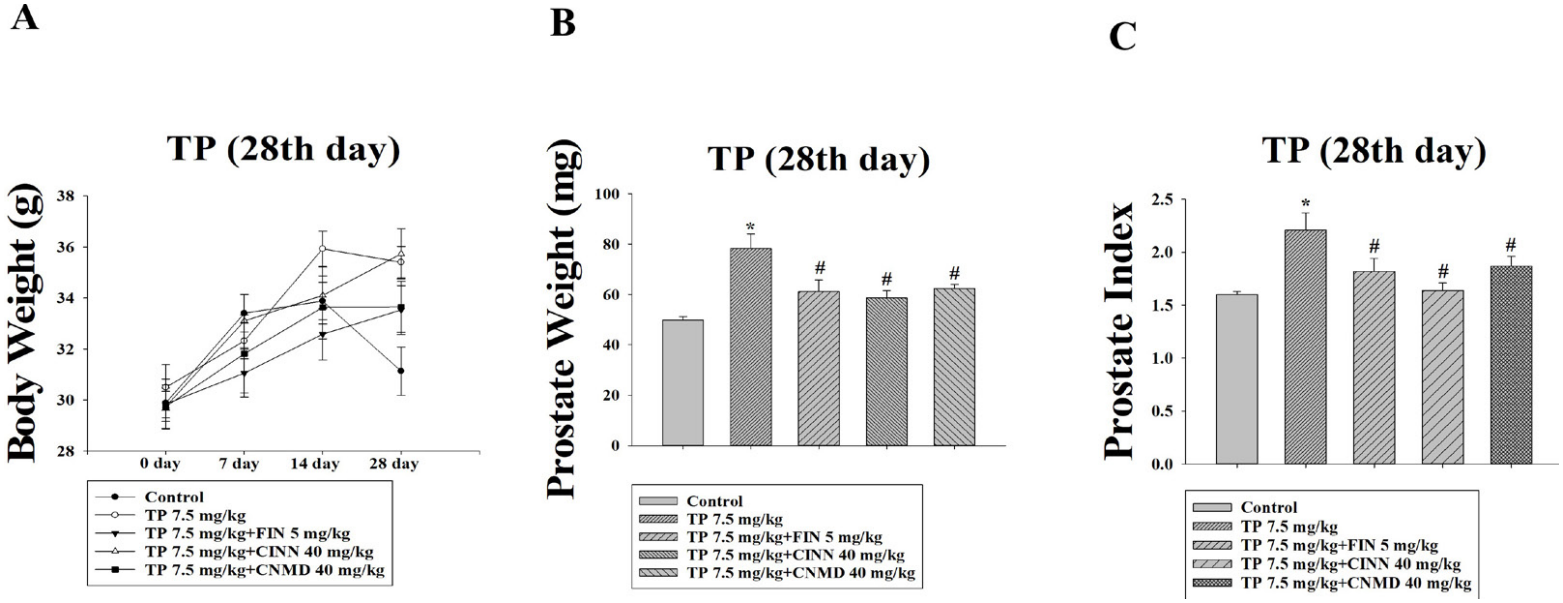
Effects of finasteride (FIN; 5 mg/kg), cinnamophilin (CINN; 40 mg/kg), and cinnamaldehyde (CNMD; 40 mg/kg) on body weight, prostate weight, and prostate index in testosterone propionate (TP; 7.5 mg/kg)-induced prostate growth in mice at day 28

Prostates were dissected, and their weights, along with the prostate index (PI), were measured. In the TP-induced group, prostate weight and PI significantly increased compared with the control group (Figure 1A-C). Treatment with cinnamophilin (40 mg/kg), cinnamaldehyde (40 mg/kg), and finasteride (5 mg/kg) attenuated the increase in PI by 93%, 55% and 65%, respectively, over 28 days (Figure 1C). 

### Histopathological evaluation

**Figure 2:**
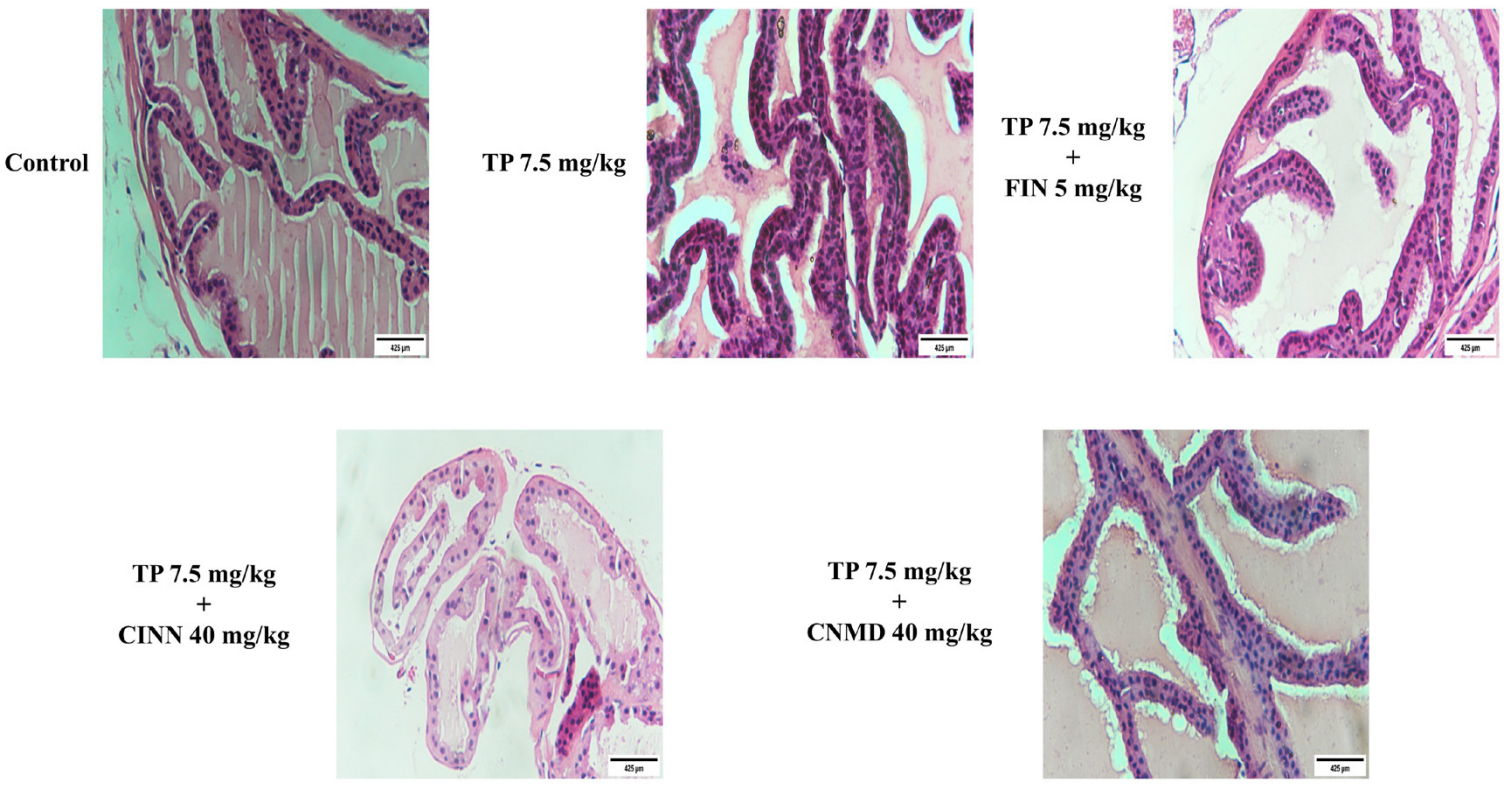
Histological analysis of finasteride (FIN; 5 mg/kg), cinnamophilin (CINN; 40 mg/kg), and cinnamaldehyde (CNMD; 40 mg/kg) in testosterone propionate (TP; 7.5 mg/kg)-induced mouse prostate growth after treatments

**Figure 3:**
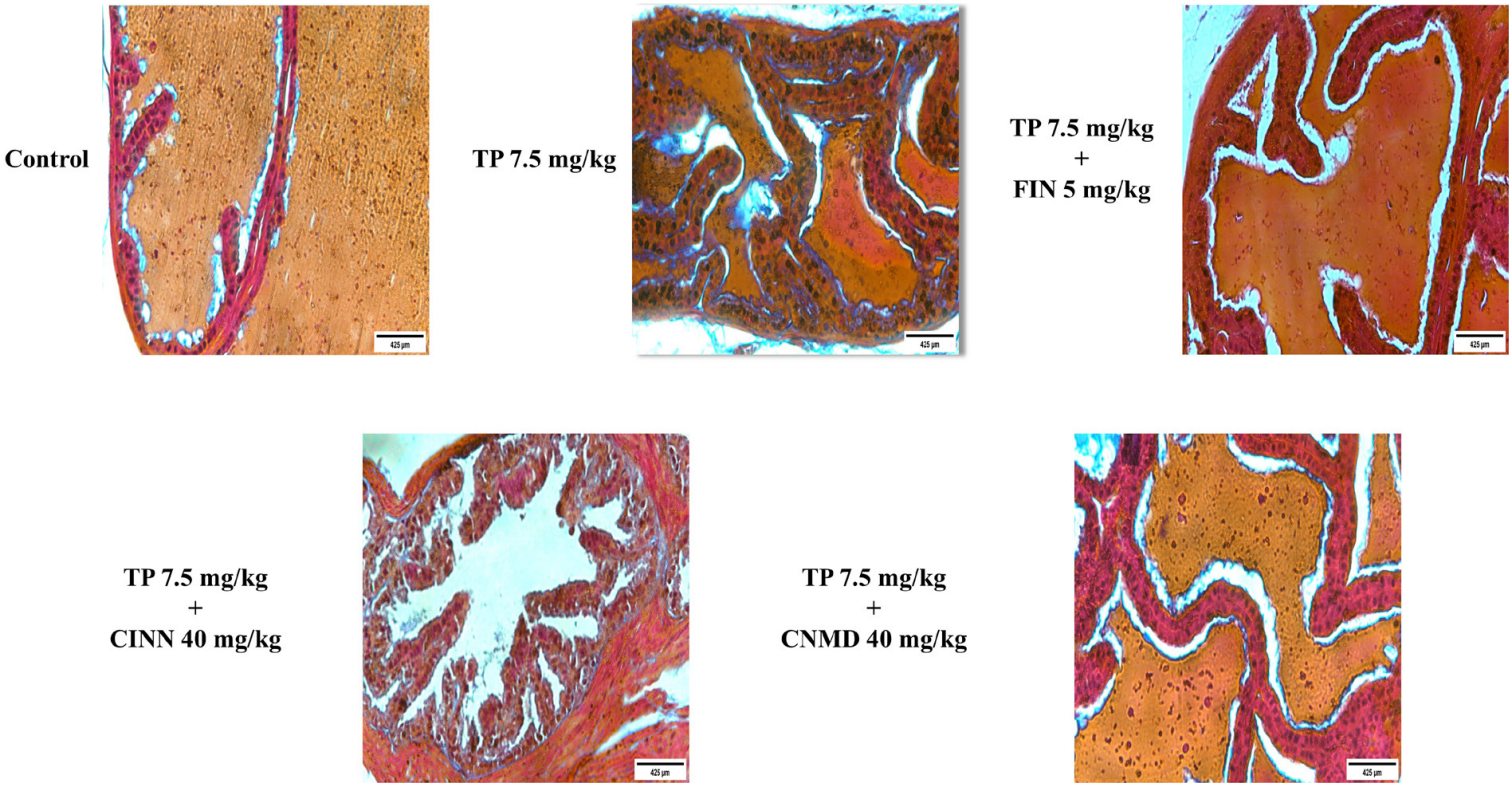
Histological analysis of finasteride (FIN; 5 mg/kg), cinnamophilin (CINN; 40 mg/kg), and cinnamaldehyde (CNMD; 40 mg/kg) in testosterone propionate (TP; 7.5 mg/kg)-induced mouse prostate growth after treatments

Mice in the TP treatment group exhibited histological changes, including thicker epithelium with irregular epithelial shapes, larger acini, decreased glandular luminal area, and tighter stroma (Figure 2) by H&E staining. However, cinnamophilin, cinnamaldehyde, and finasteride mitigated these morphological changes (Figure 2). Masson’s trichrome staining was performed to assess collagen, collagen fibers, and prostatic fibrosis; these were stained blue. Collagen deposition in the prostate of the TP-treated group was observed by Masson’s trichrome staining (Figure 3). Cinnamophilin, cinnamaldehyde, and finasteride can ameliorate the prostate fibrosis in TP-induced mice. These results indicate that cinnamophilin attenuated hyperplasia in TP-treated mice.

### Cinnamophilin reduced the viability of WPMY-1 and BPH-1 cells

**Figure 4:**
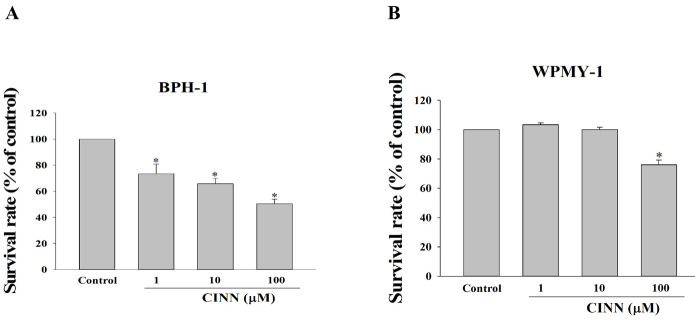
Cinnamophilin (CINN; 1-100 μM) suppressed prostate cell growth in BPH-1

The inhibitory effects of cinnamophilin on prostate cells were studied using the BPH-1 epithelial cell line (derived from BPH) and the WPMY-1 myofibroblast cell line (derived from prostate stroma). Both cell lines were treated with different concentrations of cinnamophilin (1-100 μM) for 48 hr, followed by an MTT assay. Cinnamophilin significantly inhibited cell viability in a dose-dependent manner (Figure 4A, B). At a concentration of 100 μM, the survival rates were 50.47 ± 3.48 % for BPH-1 and 76.06 ± 3.20 % for WPMY-1 cells after 48 hours of treatment.

### Cinnamophilin inhibited TP-induced prostate growth by regulating 5α-reductase type II

**Figure 5:**
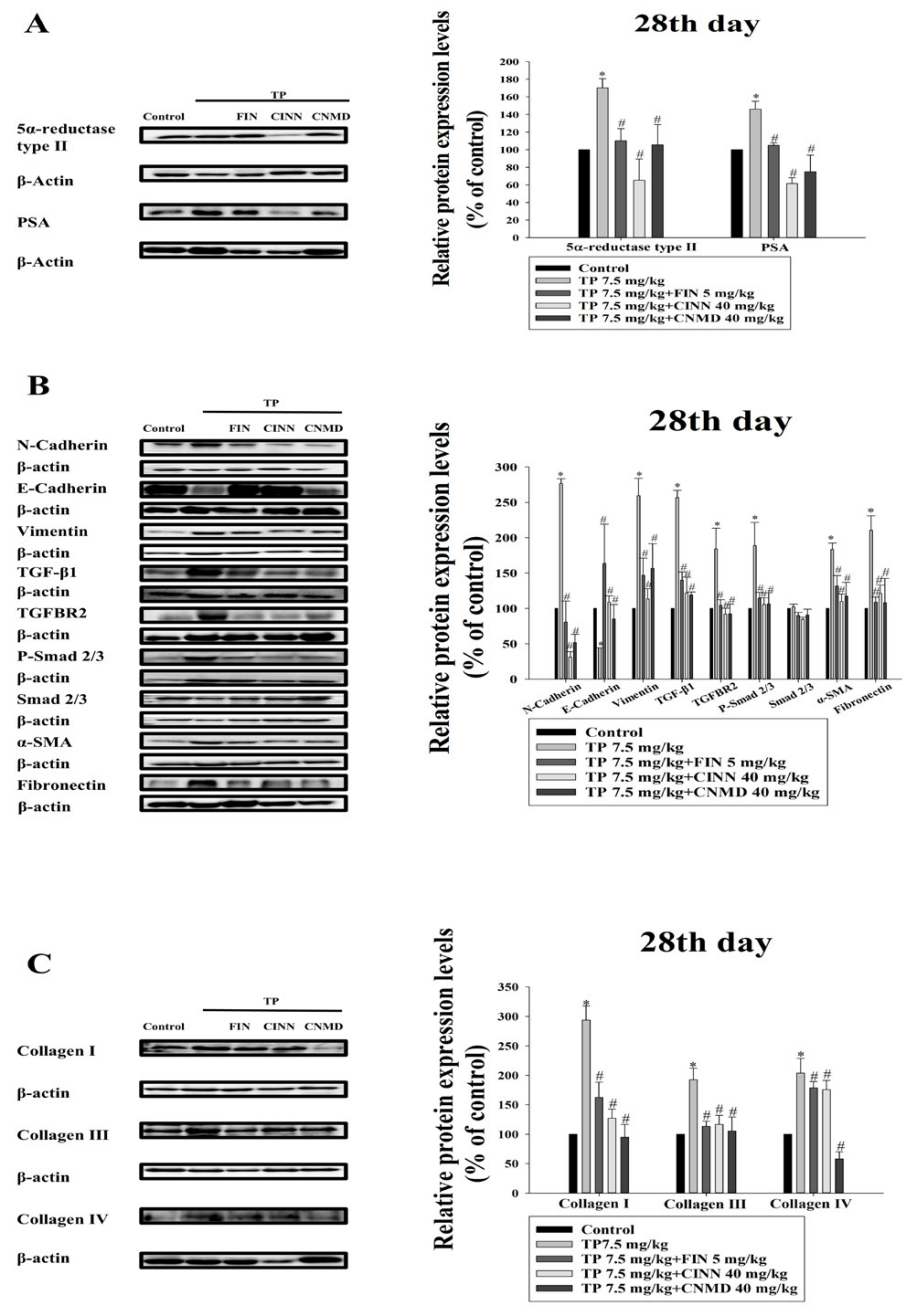
Effects of finasteride (FIN; 5 mg/kg), cinnamophilin (CINN; 40 mg/kg), and cinnamaldehyde (CNMD; 40 mg/kg) on the expression of 5α-reductase type, PSA, N-cadherin, E-cadherin, vimentin, TGF-β1, TGFBR2, p-Smad2/3, Smad2/3, α-SMA, fibronectin, collagen I, collagen III, and collagen IV in TP (7.5 mg/kg)-induced mice at day 28^th^ (A-C)

Western blot analysis was used to measure 5α-reductase type II and PSA expression in the prostates of TP-induced mice. TP treatment up-regulated the expression of both proteins (Figure 5A). However, cinnamophilin (40 mg/kg), cinnamaldehyde (40 mg/kg), and finasteride (5 mg/kg) treatments inhibited the expression of 5α-reductase type II and PSA, reversing the TP-induced effects (Figure 5A). 

### Cinnamophilin inhibited TP-induced EMT and fibrosis via the TGF-β/Smad pathway

The TGF-β/Smad signaling pathway plays a key role in EMT and fibrosis in the prostate. TP treatment up-regulated TGF-β1, TGFBR2, and P-Smad2/3 protein expression in mouse prostates. The expression of Smad2/3 was not significantly different among these groups. EMT-related mesenchymal markers (N-cadherin, fibronectin, α-SMA, and vimentin) were also up-regulated, while the epithelial marker E-cadherin was down-regulated (Figure 5B). Furthermore, fibrosis-related proteins (collagen I, III, and IV) were elevated (Figure 5C). Cinnamophilin, cinnamaldehyde, and finasteride reversed these effects (Figure 5A-C), demonstrating that cinnamophilin regulates the TGF-β/Smad signaling pathway to inhibit EMT, fibrosis, and prostate growth.

## Discussion

BPH is one of the most common conditions affecting men, particularly in older age, significantly impacting their quality of life. Factors such as androgen and metabolic syndrome can exacerbate BPH symptoms. The condition is characterized by the proliferation of epithelial, smooth muscle, and stromal cells. PSA, a protein produced by the prostate, is often elevated in the blood of patients with BPH or prostate cancer. Based on our results, an increase in the PI, prostate weight, and PSA was observed in TP-induced mice. 

Cinnamophilin, a lignan derived from the Cinnamomum genus, exhibits anti-oxidant and neuroprotective effects at doses ranging from 20–80 mg/kg in animal studies (14, 21). Based on previous findings, we used an intermediate dose of 40 mg/kg cinnamophilin in this study. Herbal extracts are rich in phytochemicals with various pharmacological activities, including anti-inflammatory, anti-oxidant, and anti-cancer properties (22, 23). Phytosterols, phenolics, and fatty acids such as β-sitosterol, lupeol, epigallocatechin-3-gallate, and oleic acid have been shown to inhibit 5α-reductase activity (24-27). In the current study, we explored the anti-proliferative effects of cinnamophilin and cinnamaldehyde against TP-induced prostate growth. A study indicated that cinnamaldehyde inhibits 5α-reductase type II and androgen receptor expression in a rat model of premalignant prostate carcinogenesis, reduces uric acid levels, and down-regulates the IL-6/JAK1/STAT3 signaling pathway in testosterone-induced prostate growth (28, 29). The high expression and activity of 5α-reductase serve an essential role in prostate enlargement. In this study, we found that cinnamophilin, cinnamaldehyde, and finasteride effectively inhibit TP-induced prostate growth by modulating 5α-reductase type II and PSA expression. Histopathological analysis revealed that cinnamophilin, cinnamaldehyde, and finasteride ameliorate the structural changes in the prostate. The anti-proliferative effects of cinnamophilin are better than those of cinnamaldehyde in an in vivo study. Thus, we further examined whether cinnamophilin can suppress prostate cell growth. According to a previous study, concentrations of cinnamophilin (4–1000 μM) were used to evaluate cell viability in malignant glioma cells (17). Cinnamophilin (20-1000 μM) significantly inhibited cell growth in glioma cell lines. The intermediate concentrations (1-100 μM) of cinnamophilin were used in the current study. Cinnamophilin (100 μM) can inhibit the prostate stroma (WPMY-1) and prostate epithelial cell (BPH-1) growth *in vitro*.

TGF-β is a multifunctional cytokine that signals through membrane-bound receptors. TGF-β, in conjunction with androgens, promotes BPH development. Activation of the TGF-β/Smad signaling pathway induces local angiogenesis, inflammation, and fibrosis, contributing to both BPH and diabetes progression (18, 30, 31). Numerous studies have identified the TGF-β/Smad signaling pathway as a key driver of EMT and fibrosis (32-34). EMT is involved in physiological processes related to wound healing, organ fibrosis, and metastasis in cancer cells (35, 36). Our findings indicate that androgen stimulation in TP-treated mice induces prostate growth via TGF-β1 production. TGFBR2 is predominant in BPH tissue (37). Cinnamophilin, cinnamaldehyde, and finasteride decreased TGF-β1, TGFBR2, and p-Smad2/3 protein expression in TP-treated mice. However, the expression of Smad2/3 was not significantly different among these groups. Since TGF-β1 is a critical mediator of EMT and fibrosis, these treatments effectively mitigated these pathological processes. A study indicated that TGF-β induces miR-223-3p expression, promoting BPH-1 cell survival and EMT (38). In TP-treated mice, TGF-β1 expression was upregulated, while E-cadherin expression was down-regulated. However, treatments with cinnamophilin, cinnamaldehyde, and finasteride decreased N-cadherin, vimentin, α-SMA, and fibronectin protein expressions, while increasing E-cadherin levels. 

The stromal components and prostatic fibrosis were notably increased in BPH tissues (5), and fibrosis, along with collagen deposition, was observed in TP-induced mice by Masson’s trichrome staining in this study. Elevated levels of collagen I, III, and IV proteins were also detected but were effectively reduced by treatments with cinnamophilin, cinnamaldehyde, and finasteride. Previously, cinnamon extract and cinnamaldehyde reduced inflammation and fibrosis in colitis by decreasing the expression of matrix metalloproteinases (39). Cinnamaldehyde attenuated fibrosis by down-regulating TGF-β-mediated ROS production and extracellular matrix components in dermal fibroblasts (40). Interestingly, cinnamaldehyde and cinnamophilin reduced fibrosis by decreasing collagen deposition and expression in this study. It is first reported that cinnamaldehyde and cinnamophilin can reduce fibrosis in the prostate. These findings suggest that blocking the TGF-β1/Smad signaling pathway represents a promising therapeutic strategy for BPH. Notably, cinnamophilin inhibited prostate growth by down-regulating TGF-β/Smad signaling pathway and reversing EMT and fibrosis.

Data are presented as mean ± SE. *P<0.05 compared with the control, #*P*<0.05 compared with the TP group.

Results were visualized by H&E staining (magnification, x 400) (bar = 425 μm).

Results were visualized by Masson's trichrome staining (magnification, x 400) (bar = 425 μm).

(A) and WPMY-1 (B) cells at 48 hr. Data are presented as mean ± SE. *P<0.05 compared with the control.

Data are presented as mean ± SE. *P<0.05 compared with the control, #*P*<0.05 compared with the TP group.

**Figure 6:**
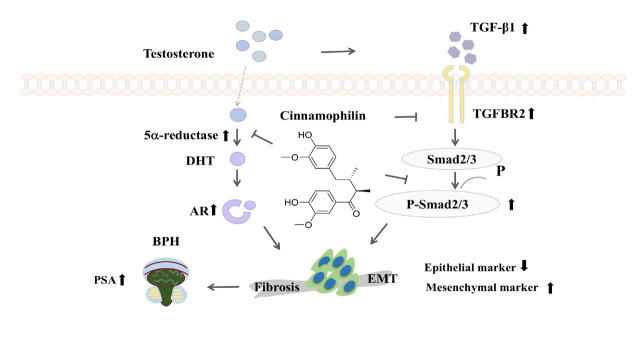
Proposed mechanism by which cinnamophili inhibits mouse 5α-reductase and TGF-β/Smad signaling pathways, thereby reversing EMT in the prostate

EMT: Epithelial-mesenchymal transition

## Conclusion

This study demonstrated that cinnamophilin inhibits prostate growth by targeting 5α-reductase and down-regulating the TGF-β/Smad signaling pathway in TP-treated mice (Figure 6). Additionally, cinnamophilin also reverses EMT and fibrosis. These results highlight the therapeutic potential of cinnamophilin for the management of BPH.

## Declaration

We have not used any AI tools or technologies to prepare this manuscript.
